# CTLA-4 Expression Plays a Role in PSC and PBC Progression

**DOI:** 10.3390/diseases8020021

**Published:** 2020-06-12

**Authors:** Phil Meister, Christian Steinke-Ramming, Mechthild Beste, Henrike Lenzen, Guido Gerken, Ali Canbay, Christoph Jochum

**Affiliations:** 1 Department of Gastroenterology and Hepatology, University Hospital Essen, 45147 Essen, Germany; christian.ramming@t-online.de (C.S.-R.); mechthild.beste@uk-essen.de (M.B.); henrike.lenzen@uk-essen.de (H.L.); meduni-kkh@ruhr-uni-bochum.de (A.C.); Guido.gerken@uk-essen.de (G.G.); 2 Department of General-, Visceral-, and Transplantsurgery, University Hospital Essen, 45147 Essen, Germany; 3 Medical Clinic, University Hospital Bochum, 44892 Bochum, Germany; 4 Department of Hepatology and Gastroenterology, Charite Berlin, 10117 Berlin, Germany

**Keywords:** PBC, PSC, immunogenetics, CTLA-4, cirrhosis

## Abstract

Background & Aims: The pathogenesis of primary biliary cholangitis (PBC) and primary sclerosing cholangitis (PSC) remains unclear. The aim of this study was to reveal certain single nucleotide polymorphisms (SNP) in genes for regulatory proteins in the immunologic pathway possibly going along with susceptibility of attaining PBC or PSC. Methods: 126 patients with either PBC or PSC with clinical and laboratory data were enrolled in the study. SNPs in three genes (CTLA-4, ICOS, and FOX-P3) which are suspected to play a key role in the autoimmune pathway were analyzed to determine allele variants. Gene expression was measured by RealTime PCR using mRNA. Results: Patients with cirrhosis had a lower number of CTLA-4 copies than patients without cirrhosis (*p* = 0.04). Accordingly, patients with lower CTLA-4 copies had a poorer recovery of gamma-glutamyltransferase (GGT) in course of their disease (−69.8 U/l vs. −176.1 U/l *p* = 0.04). Two SNP allele variants (CTLA4 rs733618 and FOXP3 rs2280883) associated with low CTLA-4 expression could be determined. Patients having both variants showed worsening of GGT (−61.7 U/l vs. −132.6 U/l, *p* = 0.04) and a trend towards a more progressive disease in terms of cirrhosis. (24% vs. 13% *p* = ns). Conclusions: Low expression of CTLA-4 is associated with a more advanced disease in patients with PBC and PSC. Furthermore, we identified two SNP allele variants (CTLA4-SNP rs733618 and FOXP3-SNP rs2280883) associated with a lower CTLA-4 expression and possibly a more severe course of the diseases. Taken together, these results provide further evidence for the involvement of the immune system in the pathogenesis of these two cholestatic liver diseases. **Lay summary**: Primary biliary cholangitis and primary sclerosing cholangitis are chronic diseases of the bile ducts. Their cause remains widely unclear, but evidence suggests the immune system plays a central role. This study shows that gene alterations connected to the immune system might play a role in the course of the disease.

## 1. Introduction

Primary biliary cholangitis (PBC) and primary sclerosing cholangitis (PSC) are chronic cholestatic liver diseases characterized by bile duct inflammation, subsequently leading to cholestasis, fibrosis, and ultimately cirrhosis and liver failure. While PBC is characterized by an immune-mediated injury to small intrahepatic bile ducts, a chronic inflammation in PSC leads to a progressive destruction of the intra- and/or extrahepatic bile ducts with segmental scarification and obstruction. The etiology of both chronic cholestatic diseases remains largely unclear. 

PBC has a clear female predominance with the highly specific anti-mitochondrial antibodies (AMA) being present in 90–95% of patients with PBC. The autoimmune basis of PBC is supported by the presence of AMA and the characteristic progressive T-cell predominant lymphocytic cholangitis [1].

Reinforcing this approach, there are several autoimmune diseases such as myasthenia gravis [2,3,4], Primary Sjögren’s Syndrome [5] and coeliac disease [6], which are linked to the same genes that are in focus regarding PBC’s possible autoimmune pathogenesis. In addition to the genetic approach there is an eye-catching clustering of cases in industrial countries and near water reservoirs, coal mining areas, and waste disposal sites [7], suggesting environmental and epigenetic factors. Consequently, incidence and prevalence rates for PBC are rising in the last decades [8].

PSC on the other hand is a male predominant disease [9], also with rising incidence and compared to PBC with a particularly high risk of cholangiocarcinoma and colorectal cancer [10].

PSC is also associated to other immune-mediated diseases such as chronic inflammatory bowel disease [9], autoimmune hepatitis, sarcoidosis, thyroid disease, and type I diabetes [11,12]. The occurrence of one or more autoimmune diseases does not influence the clinical progression of PSC [13]. There are no known specific antibodies such as AMA in PBC, although in 60% of patients with PSC elevated perinuclear anti-neutrophil cytoplasmic antibodies (pANCA) can be found [14]. Recent genome wide-association study has given evidence of genetic susceptibility for PSC with strong HLA association in genes coding the G-protein-coupled bile acid receptor 1 and the macrophage-stimulating 1 gene [15].

Despite both ailments showing aspects of autoimmune disorders, immunosuppressive therapy showed no relevant effects or even harmful adverse events in long-term-treatment [1]. For liver failure as the common end stage of both diseases, liver transplantation still is the only curative option so far, as PBC and PSC patients account to almost 10% of the European liver transplant program [16].

Considering genetic factors, immunologic aspects, and possible environmental trigger factors, the aim of this study was to reveal certain single nucleotide polymorphism (SNPs) in genes that are relevant for regulatory proteins in the immunologic pathway possibly going along with susceptibility of attaining or developing PBC or PSC and probably interfering in the disease’s course. 

Substitution of a single base pair in the sequence of a complementary double-helical DNA results in a variant of the specific gene region and is called single nucleotide polymorphism (SNP). The outcome resulting from the base substitution depends on the region within the gene the SNP is located in. A silent SNP without resulting changes in the gene’s function or also a change in encoding of the amino acid sequence are possible consequences of a SNP. 

We analyzed SNPs in three genes which have been shown to be an important factor in the mechanism of T-cell dependent inflammation [17] and therefore might play a role in the autoimmune pathway leading to development and progression of PBC and PSC. The analyzed genes included Cytotoxic T-lymphocyte-associated Protein 4 (CTLA-4), Forkhead-Box-Protein P3 (FOXP3), and Inducible T-cell COStimulator (ICOS). 

## 2. Material and Methods

### 2.1. Patients

Blood samples were collected from 126 patients with a well-documented diagnosis of PBC and PSC from the Department of Gastroenterology and Hepatology of the University Hospital Essen, Germany. The diagnosis of PBC and PSC was made according to the EASL criteria [18] and was therefore based on elevated cholestatic serum enzymes and typical cholangiographic findings in case of PSC, and presence of AMA, elevated alkaline phosphatase, and/or histological findings in case of PBC. Written informed consent was obtained from all patients prior to blood sampling. The study was approved under the identification code 09-4022 at local ethics committee and is in consistence of the Declaration of Helsinki.

Among these were 91 PBC and 35 PSC patients. 36 healthy subjects served as a control group. Both patients and the healthy cohorts were consistent with the HapMap-CEU population [19].

### 2.2. DNA Extraction and Genotyping

In order to examine allele variants, we used genomic DNA extracted from whole blood-samples using QIAamp DNA mini Kit for genomic DNA from Qiagen, Hilden, Germany. To amplify the relevant gene-section including the relevant SNPs, polymerase chain reaction (PCR) was performed using PCR Taq Plus DNAPolymerase from Quiagen according to manufacturer’s instructions. Subsequently, we performed a restriction fragment length polymorphism (RFLP) analysis. For discrimination of restriction enzymes, NEBcutter V2.0 was used [20]. In accordance with the HapMap allele constellations were grouped as major and minor variants by their predominance in the HapMap population. Furthermore, they were grouped as homozygote when only having either the major or minor allele or as heterozygote with both alleles. Results were spot-checked using DNA sequencing performed by the Institute for Human Genetics of the University Hospital in Essen, Germany. Here, SNP and primer nucleotide sequence for 10 randomly selected patients was determined and compared with presets and results. 

To examine the SNPs influence on gene regulation, gene expression was measured by RealTime PCR using mRNA, which was isolated using RNeasy Mini Kit from Qiagen following the manufacturer’s protocol. The RealTime PCR was done using Rotor-Gene SYBR Green RT-PCR Kit from Qiagen. The data were analyzed using CFX Manager Software 3.1 from BioRad (Hercules, CA, USA). Quantification was performed as relative quantification using β-Actin as internal reference.

### 2.3. Data Collection and Statistical Analysis

Clinical data were collected from the patient’s medical records. Following clinical characteristics were recorded: date of initial diagnosis, presence of cirrhosis (defined as image morphologic diagnosis) and other co-diseases. Furthermore, lab values from both the initial diagnosis and current values were noted. The difference of initial and current values is referred as “course or progression” in the following. 

Data were analyzed using SPSS 25.0 software (IBM Inc., Armonk NY, USA). The SNPs were correlated to the respective expression measured for a specific allele, clinical data and lab values. For correlation of clinical parameters, SNPs and expression, Spearman’s rank coefficient was determined. In comparison of mean values, a two-sided T-test was performed and the 95%-confidence interval (CI) given. To identify possible risk constellations two-step cluster analysis was performed. A *p*-value of <0.05 was considered statistically significant. 

## 3. Results

The PBC patients consisted of 94.6% female with a median age of 61 and 9 years of previous disease duration. 14.3% presented cirrhosis at inclusion. Mean alkaline phosphatase (AP) was 121.5 U/l and mean γ-gluutamyltransferase (GGT) was 49 U/l at inclusion. Meanwhile, PSC patients were predominantly male (57.1%) with a median age of 53 at inclusion and 7 years of previous disease duration. Cirrhosis was present in 22.9%, mean AP was 221 U/l and mean GGT was 103 U/l. Further patient characteristics are displayed in Table 1. 

The SNPs were categorized as major and minor variants in heterozygotic or homozygotic expression. There were no predominant or significantly different expressions in comparison of PBC and PSC patients in contrast to both each other and the healthy controls. 

### 3.1. Low Number of CTLA4 Copies Appears to Be Correlated with Cirrhosis and Aggravation of Liver and Cholestatic Enzymes in PBC Patients

mRNA copies measured by Real-Time PCR were correlated: CTLA4 copies showed a positive correlation with ICOS (*r* = 0.691, *p* = < 0.001) and FOX-P3 (*r* = 0.490, *p* = < 0.001) in patients with PBC and PSC. 

**Figure 1:**
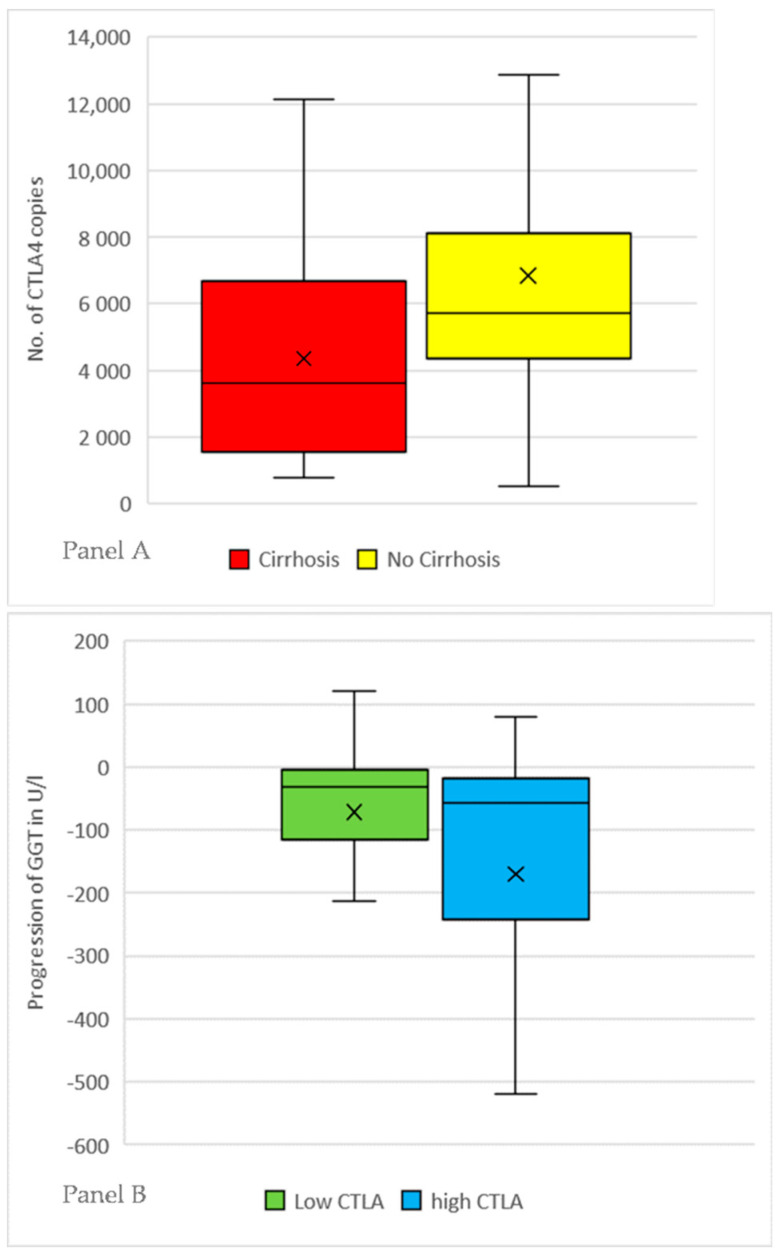
Panel **A**: Patients with primary biliary cholangitis (PBC) or primary sclerosing cholangitis (PSC) were subgrouped by presence of cirrhosis. The number of CTLA-4 copies was measured using mRNA Real-Time PCR and normalized by β-Actin as internal control. The X marks the mean value, the line inside the boxplot the median. Patients with cirrhosis have a mean number of 4359 copies, patients without cirrhosis have a mean number of 6836 copies (*p* = 0.04). Panel **B**: Patients with PBC were sorted by the determined number of CTLA-4 copies. Three equally large groups with either low, medium or high expression were formed. Patients with high and low expression were compared in the course of their disease by subtracting the lab values at initial diagnosis with the most current values for each patient. Patients with high CTLA-4 tend to have a recovery of elevated GGT-values more often [low: −69.8 U/l vs. high: −176.1 U/l *p* = 0.04]. The X marks the mean value, the line inside the boxplot the median. Patients with PSC showed the same trend without reaching statistical significance (low: −45.3 U/l vs. high: −105.4 U/l *p* = 0.08).

There was no correlation with blood cell count, INR, bilirubin, and liver transaminases in both PBC and PSC patients, especially including AP (*r* = 0.019, *p* = 0.86). Both PBC and PSC patients with cirrhosis had lower number of CTLA4 copies [mean: 4359 copies (CI:2655–6278)] than patients without cirrhosis [mean: 6836 copies(CI:5991–7746)] with a *p*-value of 0.04 in the t-test as depicted in Figure 1 Panel A. Number of ICOS-copies showed a similar result: cirrhosis [mean: 50 984(CI:25978–80493)] vs. no cirrhosis [mean: 79248(CI:68837–90431)] with a *p*-value of 0.05. 

There was a negative correlation between numbers of CTLA4 copies and course of alanine aminotransferase (ALT; *r* = −0.244, *p* = 0.02) and GGT (*r* = −0.224, *p* = 0.03) in patients with PBC, but not PSC. 

We further analyzed patients based on the number of CTLA-4 copies by forming three equally large groups with either low, medium, or high expression. The groups with low and high CTLA-4 number were compared for each disease entity separately. 

PBC patients with a low number of CTLA-4 copies showed cirrhosis more often (low: *n* = 9/30 vs. high 2/19, *p* = 0.02) and weaker recovery of GGT in course of their disease, defined as difference between initial diagnosis and the most current values. (low: −69.8 U/l vs. high: −176.1 U/l, *p* = 0.04). A significant difference in the mean length of disease was also observed (low: 13.7 years vs. high: 8.7 years, *p* = 0.01). Results are illustrated in Figure 1 Panel B. 

In the PSC cohort patients with low CTLA copies had lower thrombocyte count (low: 214/nl vs. high: 280/nl, *p* = 0.05) and a trend towards worse GGT recovery (low: −45.3 U/l vs. high: −105.4 U/l *p* = 0.08), but no significant difference in current GGT (low: 118 U/l vs. high: 221 U/l, *p* = 0.10), AP (low: 208 U/l vs. high: 268 U/l, *p* = 0.18), presence of cirrhosis (low: 5/21 vs. high: 4/17, *p* = 0.93) and mean length of disease (low: 8.2 years vs. high: 7.1 years, *p* = 0.67). 

### 3.2. Certain SNP-Haplotypes of CTLA4- and FOX-P3 Gene Are Correlated with a Lower Expression of CTLA4

The two-step genome cluster analysis for both PBC and PSC patients showed a medium cohesion and separation by the investigated SNPs. The determined cluster did not have any influence on number of copies or clinical data. 

Furthermore, we analyzed each SNP haplotype on its influence on the corresponding mRNA expression. Both CTLA4 SNP rs733618 and FOXP3 SNP rs2280883 in their homozygotic major variants (TT) are associated with a reduced number of CTLA4 copies. Rs733618 as homozygotic major has a mean of 5729 CTLA4 copies, while the heterozygotic variant has a mean of 9429 copies (*p*= 0.001); rs2280883 with the homozygotic major shows 4840 CTLA4 copies versus 6491 copies in the heterozygotic variant (*p* = 0.008).

Patients with certain SNP-haplotypes present with advanced liver disease and worsening of cholestatic enzymes more often.

**Figure 2:**
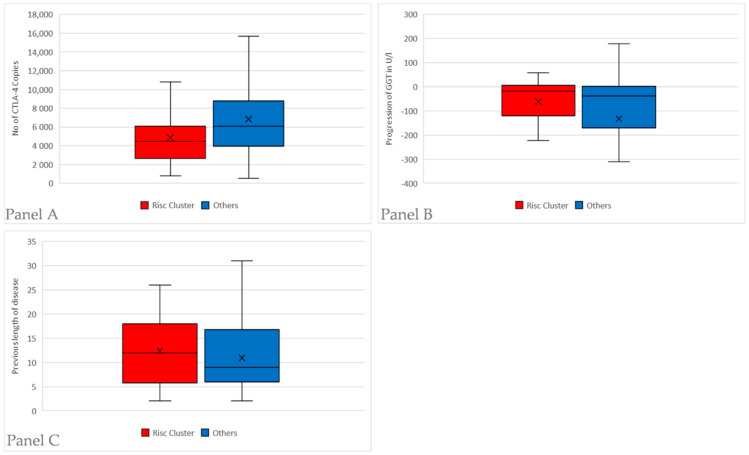
All patients were subgrouped by their allele variants in the two SNPs CTLA4 rs733618 and FOXP3 SNP rs2280883. If they were both homozygotic with the major variant (TT), defined as the predominant allele in the HapMap-population, they were included in the “risk cluster” (*n* = 44). Afterwards, they were compared with the remaining patients with a different allele variant in the two SNPs (*n* = 81). Panel **A**: Comparison of the number of CTLA-4 copies measured by mRNA Real-Time PCR and normalized using β-Actin as internal control. Patients in the “risk cluster” have lower number of CTLA-4 copies (5729 vs. 9429, *p* = 0.001). Panel **B**: Comparison of GGT progression by subtracting the lab values at initial diagnosis with the most current values for each patient. Patients in the “risk cluster” have a weaker recovery of GGT in course of their disease (−61.7 U/l vs. −132.6 U/l, *p* = 0.04). Panel **C**: Comparison of previous disease length for the “risk cluster” and the remaining patients. There is no significant difference in disease duration (12.4 years vs. 10.9 years, *p* = 0.3).

**Figure 3:**
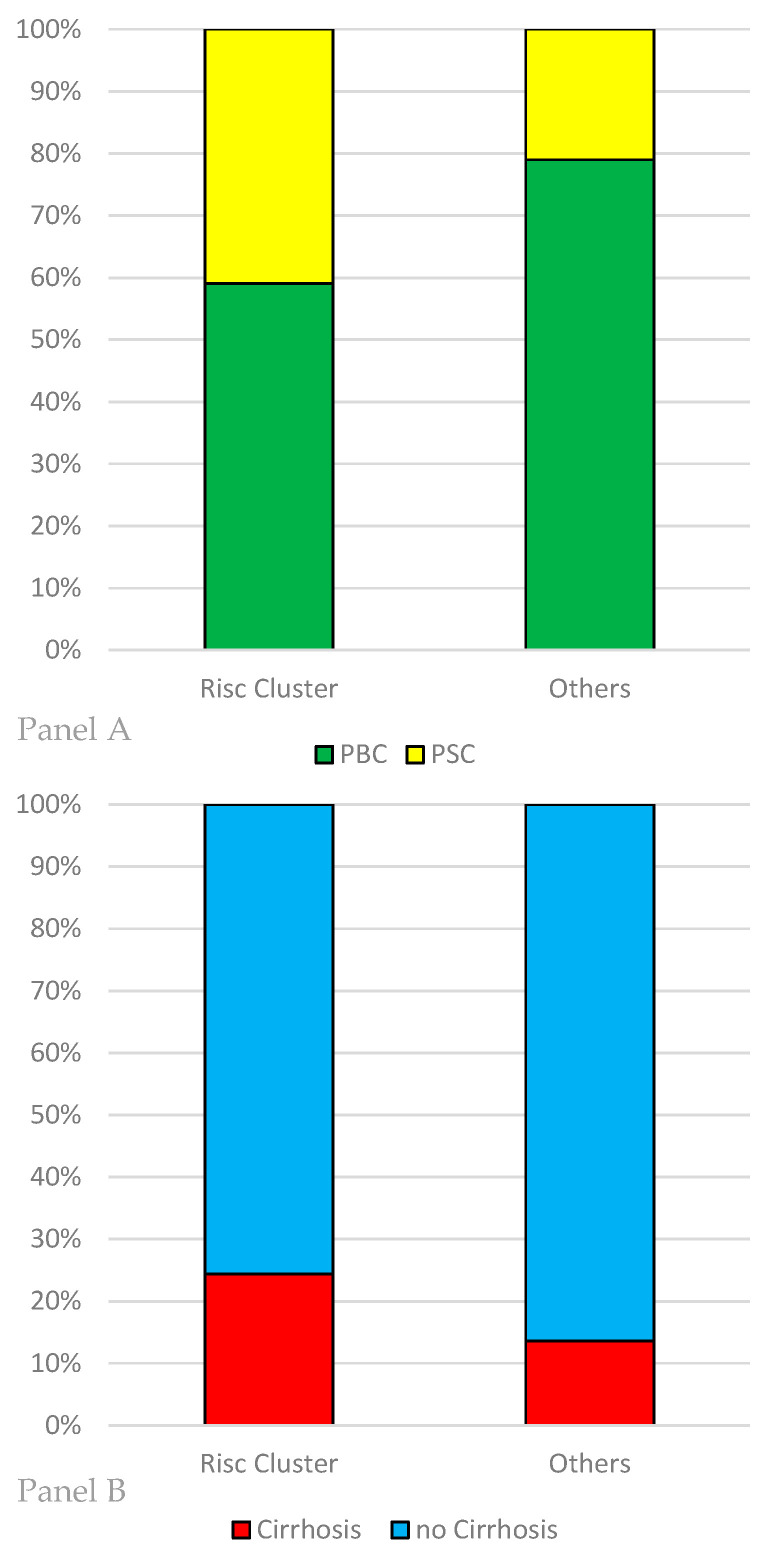
Characteristics of the subgroups formed by their allele variants in the two SNPs CTLA4 rs733618 and FOXP3 SNP rs2280883. If they were both homozygotic with the major variant (TT), defined as the predominant allele in the HapMap-population, they were included in the “risk cluster” (*n* = 44). Afterwards, they were compared with the remaining patients with a different allele variant in the two SNPs (*n* = 81). Panel **A**: Distribution of the disease entities in the risk cluster and the other patients. PSC appears more often in the “risk cluster” (*n* = 18/44; 40% vs. *n* = 17/81, 21%, *p* = 0.03). Panel **B**: Patients in the “risk cluster” tend to present cirrhosis more often in our cohort (*n* = 10/41, 24% vs. *n* = 11/81, 13%, *p* = 0.17).

We compared the patients having CTLA4 SNP rs733618 and FOXP3 SNP rs2280883 in their homozygotic major variants (TT) (*n* = 44) with the other patients (*n* = 81). The homozygotic patients had lower number of CTLA−4 copies (4870 vs. 6848, *p* = 0.003). Progression of GGT, defined as difference between initial diagnosis and current value, showed weaker recovery than in the other patients (−61.7 U/l vs. −132.6 U/l, *p* = 0.04). There is no significant difference in mean AP levels of these patients (158.1 U/l vs. 189.7 U/l, *p* = 0.13) PSC is more often the relevant diagnosis in this group than in the other allele variants (*n* = 18/44; 40% vs. *n* = 17/81, 21%, *p* = 0.03). Also, cirrhosis tends to occur more often (*n* = 10/41, 24% vs. *n* = 11/81, 13%), but this difference is not statistically significant (*p* = 0.17). There is no significant difference in previous disease duration (12.4 years vs. 10.9 years, *p* = 0.3). The characteristics of this “risk cluster” having both CTLA4 SNP rs733618 and FOXP3 SNP rs2280883 in their homozygotic major variants are depicted in Figure 2 and Figure 3. 

## 4. Discussion

The pathogenesis of the two cholestatic liver diseases PBC and PSC is still widely unknown. Firstly, we observed that the number of investigated RNA copies is strongly adherent. While possible technical reasons in course of the RealTimePCR measuring must be considered, the idea of mutual regulation in a system as complex as the immune system appears likewise reasonably.

### 4.1. Lack of CTLA-4 as Inhibitory Marker Is Linked to a More Advanced Disease in PBC & PSC Patients

In our study the cytotoxic T-lymphocyte-associated protein 4 (CTLA-4) appears to play a role in the disease. We could determine a significant correlation between number of CTLA-4 copies, cirrhosis, and course of GGT in time between inclusion and diagnosis, maybe in context of disease length. This might be interpreted that patients having fewer CTLA-4 copies tend to have a more advanced disease. A reasonable explanation for the reduced number of CTLA-4 mRNA copies is the immune suppression caused by cirrhosis; in others words, a longer disease may cause a downregulation of CTLA-4 expression. Interestingly CTLA-4 itself is characterized as an inhibitory protein [21,22] implying that the reduced number of CTLA-4 copies rather goes along with a more active immune system. Therefore, the more intriguing explanation is, that the more frequent presence of cirrhosis and worse course of GGT is being caused by the low number of CTLA-4 copies, as the immune system lacks CTLA4-transmitted inhibition and might attack the bile ducts more fiercely. Nevertheless, these correlations in either interpretation underline the role the immune system plays in both PBC and PSC and is another step into understanding the diseases.

### 4.2. We Describe an Association of the CTLA4 SNP rs733618 and the FOXP3 SNP rs2280883 with PBC and PSC for the First Time

Including our immunogenetic investigations we identified the CTLA4 SNP rs733618 and the FOXP3 SNP rs2280883 in their homozygotic major variants (TT) being associated with reduced number of CTLA-4 copies. The first mentioned SNP rs733618 is located on chromosome 2 upstream of CTLA-4 and is part of the gene’s activator sequence. FOXP3 SNP rs2280883, located on the X chromosome, is part of the gene’s intron [18]. Their location suggests both SNPs are synonymous for the genes’ amino acid sequence but might have an influence on the regulation of both genes.

Relating to our findings previous studies identified CTLA4 SNP rs733618 to be significantly associated with myasthenia gravis [23,24,25]. An association with PBC or PSC and rs733618 has not been demonstrated yet. Regarding FOXP3 SNP rs2280883 a possible relation between systemic sclerosis in female patients as well as a potential association with hepatitis B-related hepatocellular carcinoma were described [26,27]. A linkage between PBC or PSC and rs2280883 has not been reported yet.

If both the SNP variants are present, patients not only have a reduced number of CTLA-4 mRNA copies, but also have cirrhosis more often and course of GGT tends to be worse independent from previous disease length. Once more this suggests a relevant role of CTLA-4 activity in development and progression of the disease.

### 4.3. The Role of Epigenetic and Immunogenetic Features in PBC and PSC

This goes in line with findings that the disruption of several genes involved in the regulation of immune response [i.e. TGF-β II, IL-2 receptor or anion exchanger 2(AE2)] leads to histologic and serologic changes as found in PBC [28]. Similarly, the suggested association with HLA haplotype B8 DR3 [13] furthermore supports the immunogenetic conception in PSC. A genome-wide analysis of PSC patients in comparison with healthy controls was performed in 2010 [15], where G-protein-coupled bile acid receptor 1 and macrophage-stimulating 1 gene could be identified as possible disease genes. A similar genome-wide association analysis was performed for PBC patients [29]. A CTLA-4 involvement has not been proven for both disease entities, yet. The previously mentioned study and other genome-wide studies were aimed on characterizing genetic susceptibility for cholestatic liver diseases, while genes affecting the course of the disease were not considered due to the studies’ design. In synopsis of our results, reduced number of CTLA4 copies might promote disease progression, possibly without participating in general susceptibility. Both allele variants of the “risk cluster” are rather common in the general population but still appear to play a role in a rare disease, which underlines they are only one of many factors influencing susceptibility and progression of the disease, while other genetic and environmental factors need to come together for the disease to fully develop.

The relevance of the individual epigenetic features and their role in the disease’s severity regarding PBC is underlined by findings among monozygotic twins. It showed that progression and disease severity varied although the age of disease onset and subsequently disease duration were similar [30].

All these features might help in the approach to both diseases. A set of multiple immunogenetic variants could possibly foresee the severity and outcome of the disease or even test the possibility of occurrence. Also, every marker is a possible target for future therapies trying to influence outcome and prognosis. As our study has a very heterogeneous patient collective and accordingly only limited statistical power our results should be verified on a larger cohort. We encourage the investigation of further genetic markers possibly connected to the susceptibility of attaining or developing PBC or PSC.

## 5. Conclusions

In conclusion, out study could show that the cytotoxic T-lymphocyte-associated Protein 4 (CTLA-4) appears to play a role in the autoimmune pathway connected to PSC, respectively PBC. Having both the CTLA4 SNP rs733618 and the FOXP3 SNP rs2280883 in their homozygotic major variants (TT) corresponds to a lower number of CTLA-4 copies and therefore to a possibly more severe disease.

Our results once more underline the importance of immunogenetics in the pathogenesis of both diseases.

## Figures and Tables

**Table 1:** Collective Characteristics: This table gives an overview of the examined cohort and their characteristics. IQR = Interquartile range, UDCA = Ursodeoxycholic acid, AST = Aspartat-Aminotransferase, ALT = Alaninaminotransferase, GGT = Gamma-Glutamyltransferase, AP = Alkaline phosphatase.

Characteristic	Overall (*n* = 126)	PBC (*n* = 91)	PSC (*n* = 35)
Male, *n* (%)	25 (19.8%)	5 (5.4%)	20 (57.1%)
Age at inclusion, median (IQR)	59 (49–70)	61 (51–70)	53 (48–67)
Age at diagnosis, median (IQR)	45 (35–52)	47 (38.5–52.25)	32 (26.5–44)
Years with disease, median (IQR)	6 (3–12)	9 (5–18)	7 (3.5–11)
Inflammatory bowel disease, *n* (%)	17 (13.5%)	1 (1.1%)	16 (45.7%)
UDCA therapy, *n* (%)	113 (89.7%)	80 (87.9%)	33 (94.2%)
Cirrhosis, *n* (%)	22 (17.5%)	13 (14.3%)	8 (22.9%)
AST (U/l), median (IQR)	33 (22–52.5)	28 (20.5–40)	48 (36–100)
ALT (U/l), median (IQR)	32 (22.5–46)	29.5 (22–46.5)	39.5 (31–93)
GGT (U/l), median (IQR)	63 (27–145.5)	49 (24–111)	103 (64–180)
AP (U/l), median (IQR)	133 (95.5–219)	121.5 (93–181.5)	221 (131–338)
Platelets (×10^9/l), median (IQR)	252 (200–307)	254 (203–309)	240 (183–307)
Bilirubin (mg/dl), median (IQR)	1 (0.5–1)	0.6 (0.4–0.8)	0.5 (0.2–1.2)

## Abbreviations

PBC	primary biliary cholangitis
PSC	primary sclerosing cholangitis
AMA	anti-mitochondrial antibodies
pANCA	perinuclear anti-neutrophil cytoplasmic antibodies
HLA	human leukocyte antigen
SNP	single nucleotide polymorphism
CTLA4	Cytotoxic T-lymphocyte-associated Protein 4
FOX P3	Forkhead-Box-Protein P3
ICOS	Inducible T-cell COStimulator
PCR	polymerase chain reaction
RFLP	restriction fragment length polymorphism
CI	confidence interval
AP	alkaline phosphatase
GGT	γ-glutamyl transferase
ALT	alanine transferase
INR	international normalized ratio
